# Direct Growth of Al_2_O_3_ on Black Phosphorus by Plasma-Enhanced Atomic Layer Deposition

**DOI:** 10.1186/s11671-017-2016-x

**Published:** 2017-04-20

**Authors:** B. B. Wu, H. M. Zheng, Y. Q. Ding, W. J. Liu, H. L. Lu, P. Zhou, L. Chen, Q. Q. Sun, S. J. Ding, David W. Zhang

**Affiliations:** 1 The Key Laboratory of Food Colloids and Biotechnology, Ministry of Education, School of Chemical and Material Engineering, Jiangnan University, Wuxi, 214122 Jiangsu China; 2 State Key Lab ASIC & Syst., Department Microelectronics, Fudan University, Shanghai, 201203 China

**Keywords:** Black phosphorus, Plasma-enhanced atomic layer deposition, Oxygen plasma, Al_2_O_3_

## Abstract

Growing high-quality and uniform dielectric on black phosphorus is challenging since it is easy to react with O_2_ or H_2_O in ambient. In this work, we have directly grown Al_2_O_3_ on BP using plasma-enhanced atomic layer deposition (PEALD). The surface roughness of BP with covered Al_2_O_3_ film can reduce significantly, which is due to the removal of oxidized bubble in BP surface by oxygen plasma. It was also found there is an interfacial layer of PO_*x*_ in between amorphous Al_2_O_3_ film and crystallized BP, which is verified by both X-ray photoelectron spectroscopy (XPS) and transmission electron microscopy (TEM) measurements. By increasing temperature, the PO_*x*_ can be converted into fully oxidized P_2_O_5_.

## Background

Two-dimensional (2D) semiconductor materials, such as graphene [1, 2], MoS_2_ [3, 4], WSe_2_ [5], WS_2_ [6], MoTe_2_ [7, 8], SnSe_2_ [9], and black phosphorous (BP) [10–18], have been widely studied for the potential applications in the next generation devices including field emitters [10–12], gas sensors [13, 14], solar cells [17], field-effect transistors [18], optoelectronics [19], and light-emitting diodes [20]. Among these 2D materials, BP is found to be more thermodynamically stable under the ambient conditions [14]. BP is an anisotropic lamellar semiconductor and has a direct band gap of ~0.3–1.5 eV from the bulk to monolayer structure [21–25]. BP transistors also have high carrier mobility up to 1000 cm^2^/(V · s) at room temperature, on/off current ratio over 10^4^ [18, 26]. Thus, BP may be a promising candidate for electronic and optoelectronic device applications [27]. However, exfoliated BP films will degrade in the air ambient owing to the possible reactions between BP and the adsorbed water and oxygen, thus leading to a significant reduction of BP carrier mobility and on/off current ratio [27, 28]. Therefore, efficient and reliable isolation/passivation layers are necessary for BP to preserve its inherent structure and property.

So far, many efforts on passivation for BP have been made, such as an encapsulation layer for BP with boron nitride (*h*-BN) [29–33], creating saturated P_2_O_5_ on BP surface [34–36], atomic layer deposited dielectric capping [27, 37–40]. Nevertheless, *h*-BN passivation requires complicated environmental conditions and has extremely low yield [29–33]. The P_2_O_5_ which was created on BP surface provides only the short-time protection since the oxygen and moisture in the air can erode it slowly [34–36]. It is also quite tough for atomic layer deposition (ALD) to form a high-quality and uniform top dielectric film on BP because of no dangling bonds. Therefore, it is important to prevent BP-based devices from degradation in the air ambient by covering a protective oxide dielectric. Moreover, uniform and reliable dielectrics are also essentially needed for the top-gate BP devices.

In this work, uniform Al_2_O_3_ was directly grown on BP flakes using plasma-enhanced atomic layer deposition (PEALD) with the help of O_2_ plasma as an oxygen precursor, instead of H_2_O, to react with trimethylaluminum (TMA) [41]. The composition and properties of the interfacial layer between Al_2_O_3_ and BP have been examined by physical characterizations, and the mechanisms behind are analyzed.

## Methods

Few-layer BP (purity: 99.998%, Smart Elements) was transferred onto a Si substrate with thermally grown 285 nm SiO_2_ using a micromechanical method with polydimethylsiloxane (PDMS) [1, 28, 42]. Prior to transfer, the SiO_2_ surface was ultrasonically cleaned in turn by acetone and isopropyl alcohol (IPA) and piranha solutions for 10 min each, followed by 100% O_2_ annealing at 500 °C for 3 min using rapid thermal annealing (Annealsys As-One). The optical images of BP were acquired by an optical microscope (BA310Met, Motic) equipped with a camera. Raman spectroscopy measurements were performed using LabRam-1B (the Raman spectral resolution was 1.1 cm^−1^) with an excitation wavelength of 532 nm at room temperature in the air ambient. The laser power was maintained at around 0.5 mW to prevent any heating-induced damage during the measurement. Al_2_O_3_ films on BP were deposited using PEALD with O_2_ plasma and TMA precursors at different temperatures. The freshly exfoliated BP samples were transfer to Picosun 200R ALD chamber (the vacuum pressure was 12 hPa). PEALD of Al_2_O_3_ was carried out with successive cycles of O_2_ plasma and TMA precursors, with an Ar carrier gas (99.9997%, Airgas) at a flow rate of 300 sccm, 15 s pulse + 10 s Ar purge time for O_2_ plasma (The O_2_ plasma RF Power was 2000 W), 0.1 s pulse + 5 s Ar purge time for TMA (the precursor temperature was 18 °C) at a substrate temperature of 200 °C. The surface and interfacial properties of Al_2_O_3_ on BP were physically characterized using atomic force microscopy (AFM, Dimension Edge, Bruker), XPS (AXIS ULDLDTRA, Shimadzu), and TEM (Tecnai G^2^ F20 S-TWIN, FEI) measurements at room temperature.

## Results and Discussion

**Fig. 1:**
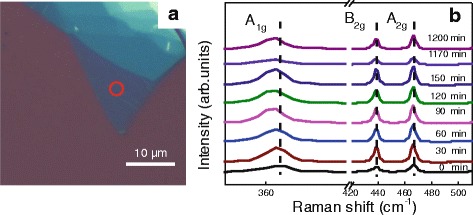
**a** Optical microscope image of the BP sample acquired by Raman measurements. **b** Raman spectra of pristine BP for different exposure time. All Raman measurements were done in the air ambient with the same laser excitation

**Fig. 2:**
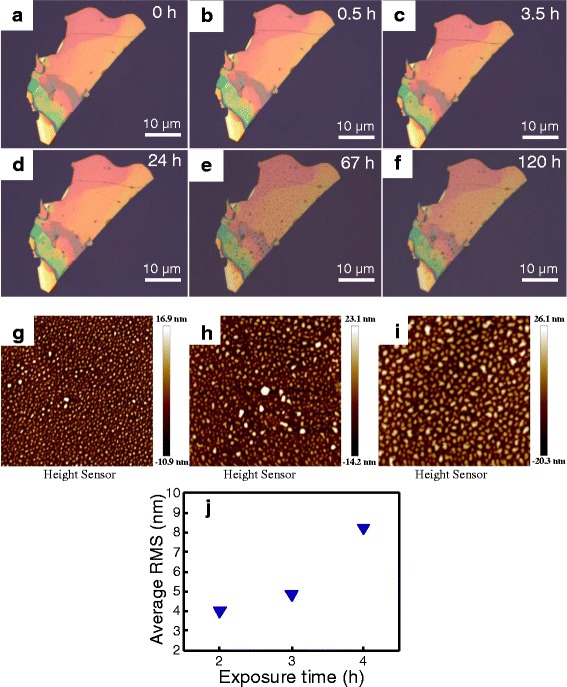
**a-f** Optical images of accelerated BP degradation on SiO_2_/Si exposed to air for different time. **g-i** AFM images of BP flake exposed to the air ambient for 2, 3, and 4 h. All three BP samples for AFM measurements were taken from the same batch. The average thickness of BP in **g–i** was 130 nm. **j** The average RMS roughness of BP samples versus the exposure time, the same samples as shown in **g–i**

Figure 1a shows the optical image of transferred BP sample prepared by mechanical exfoliation from its bulky crystalline. The Raman spectra of thin-layer BP, as denoted by a red circle in Fig. 1a, were examined as a function of exposure time in the air ambient at room temperature, as shown in Fig. 1b. It is noted that all Raman spectra measured in Fig. 1b are calibrated using a Si peak of 520 cm^−1^. It can be clearly seen one out-of-plane modes (A_1g_) and two in-plane modes (A_2g_ and B_2g_) in thin-layer BP [43]. Both A_2g_ and B_2g_ peak positions keep almost unchanged. While for A_1g_ mode, it has redshifted as the exposure time goes up to 30 min and then seems to be stable up to 20 h. This may be attributed to the oxidation of surficial BP in the initial stage and a relatively saturation of oxidation up to 20 h. This is evidenced by the time evolution of BP surface morphology examined by optical microscopy, as shown in Fig. 2a–f. It was markedly observed that BP flake exposed to the air ambient degrades as the exposure time extended and then exhibited a fare rough BP surface with bubbles, as presented in Fig. 2d–f. Figure 2g–i shows AFM images of exfoliated BP flake exposed to the air ambient for 2, 3, and 4 h, respectively. All three BP samples for AFM measurements were taken from the same batch and their RMS roughness is summarized in Fig. 2j. The RMS roughness of BP surface increases as the exposure time increases, indicating the formation of oxidative phosphorus species.

**Fig. 3:**
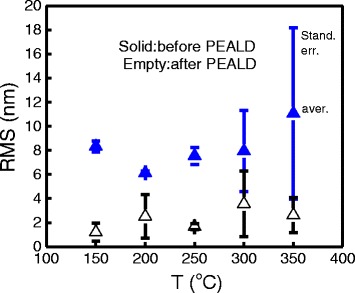
RMS roughness of BP samples before and after PEALD deposition at various temperatures. For each deposition temperature, more than five samples were measured for an accurate assessment

To evaluate the surface quality of Al_2_O_3_ film on thin-layer BP, its roughness of RMS was compared quantitatively at different deposition temperatures, as shown in Fig. 3. The corresponding data were summarized in Table 1. Note that more than five samples were measured for each temperature. It was observed that prior to PEALD, the average RMS of thin BP surface is larger than 6 nm, large scattering is due to sample-to-sample variations; however, the RMS reduces to ~3 nm after 100 cycles Al_2_O_3_ deposition. This infers that O_2_ plama as an oxygen precursor can effectively etch the oxidized bubbles in the top-layer BP thin film, thus leading to a significant reduction of RMS, while H_2_O as an O source may not have this benefit (discuss later).

**Table 1:** The RMS roughness of BP samples before and after PEALD at different temperatures

Temperature (°C)	The average roughness (before/after) (nm)	Standard deviation (before/after) (nm)
150	8.33/1.20	0.46/0.76
200	6.13/2.52	0.16/1.80
250	7.55/1.66	0.71/0.24
300	7.95/3.56	3.34/2.73
350	11.05/2.63	7.10/1.44

**Fig. 4:**
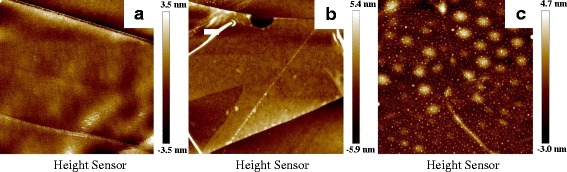
AFM images of Al_2_O_3_/BP samples with different deposition recipes at 200 °C. **a** 100 cycles Al_2_O_3_ on BP grown by PEALD with the pretreatment of 20 cycles O_2_ plasma. **b** 100 cycles Al_2_O_3_ on BP grown by PEALD without any pretreatment. **c** 100 cycles Al_2_O_3_ on BP grown by ALD with H_2_O as an oxygen precursor

To understand the impact of the pretreatment of O_2_ plasma and different oxygen precursors on Al_2_O_3_ growth in freshly exfoliated BP samples, Al_2_O_3_ deposition on BP was realized by three approaches: (1) 20 cycles O_2_ plasma pretrement + 100 cycles TMA/O_2_ plasma, (2) 100 cycles TMA/O_2_ plasma, and (3) 100 cycles TMA/H_2_O, as shown in Fig. 4a, b, and c, respectively. Figure 4a, b depicts AFM images of the 100 cycles Al_2_O_3_ grown on BP samples by PEALD with and without an oxygen plasma pretreatment, respectively. Using PEALD for Al_2_O_3_ growth in BP flakes, it has demonstrated a highly uniform surface morphology of Al_2_O_3_/BP. The average RMS roughness of Al_2_O_3_/BP samples prepared by PEALD is only 0.4 nm regardless of an oxygen plasma pretreatment, as shown in Fig. 4a, b. For freshly exfoliated BP samples, PEALD (with and w/o pretreatment) can achieve a good uniformity and coverage of Al_2_O_3_ films. While for BP samples exposed to the air ambient for certain time, 4 h for example, PEALD with O_2_ plasma pretreatment is much preferred. O_2_ plasma pretreatment can create enough nucleation sites for ALD growth. On the other hand, it also has an “etching” effect for thinning BP samples. O_2_ plasma may penetrate the PO_*x*_ layer and oxidize the underlying BP, then increase the thickness of PO_*x*_ layer [35]. On the contrary, Al_2_O_3_ films on freshly exfoliated BP grown by ALD with H_2_O as an oxygen precursor nucleate to an isolated “island” and exhibit a remarkable nonuniform surface profile, resulting in a large RMS roughness of 0.8 nm, as shown in Fig. 4c. It is attributed to the insufficient dangling bonds or nucleation sites in BP surface for ALD growth with H_2_O as an oxygen precursor [37]. It is worthwhile to mention that BP flake was covered uniformly by Al_2_O_3_ film and can prevent O_2_ or H_2_O in air ambient further reacting with BP, thus protected BP from degradation. Otherwise, the uncovered portions of BP surface may react with H_2_O and O_2_ to produce many bubbles, as shown in Fig. 4c.

**Fig. 5:**
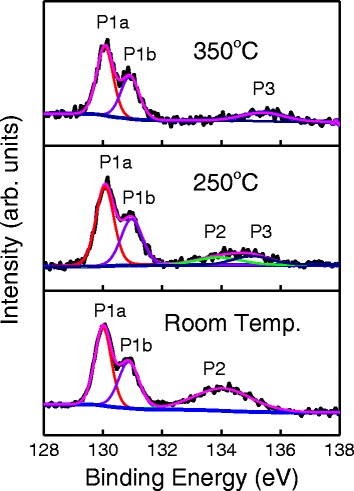
P 2p XPS spectra of the interface between Al_2_O_3_ and BP of samples at different deposition temperatures. P1a(P2p_3/2_)and P1b(P 2p_1/2_) represent phosphorus bonded to phosphorus; P2 and P3 represent the different oxidative species of PO_*x*_ and P_2_O_5_, respectively

Next, chemical analysis of the interfacial characteristics near BP film was examined by XPS characterizations. Figure 5 shows photoelectron spectroscopy measurements of the P 2p core level at different deposition temperatures. Middle and top panels present the P 2p core level after growth of 100 cycles Al_2_O_3_ by PEALD at 250 and 350 °C, respectively. The peaks which labeled as P1a and P1b correspond to P-P bonds. The phosphorus core level contains only the characteristic doublet representing the P 2p_3/2_ (P1a) and P 2p_1/2_ (P1b) orbitals with peak positions of 130.06 eV and 130.92 eV, respectively, consistent with the report [34]. Two peaks of P2 and P3 were observed at 134.07 and 135.03 eV, corresponding to the PO_*x*_ and P_2_O_5_, respectively, as shown in the middle panel. It can be seen that the PO_*x*_ peak locates at 134.07 eV, smaller than that of reported P_2_O_3_ (134.2 eV) [44], which may be caused by less O concentration in the interfacial PO_*x*_ layer. The P3 represents the most dominant oxide component and appears at 135.03 eV, in good agreement with the reported P_2_O_5_ binding energies which are between 135.0 eV [45] and 135.15 eV [37]. When the temperature goes up to 350 °C, interestingly, P2 peak disappears. This is due to the conversion from PO_*x*_ to P_2_O_5_, with the help of reactivity of O_2_ plasma at high temperatures. However, there is no P3 peak for natively oxidized BP at room temperature and its peaks of P1a; P1b and P2 locate at 130.06 eV(P 2p_3/2_), 130.87 eV(P 2p_1/2_), and 134.05 eV, respectively. The absence of P3 peak is due to low temperature or insufficient exposure time for the formation of fully oxidative top layer, which may prevent PO_*x*_ from converting into P_2_O_5_ film.

**Fig. 6:**
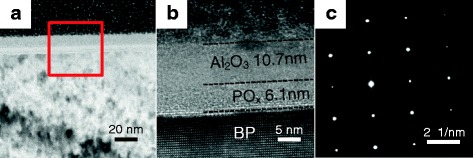
**a** Low-magnification TEM image of Al_2_O_3_/BP sample fabricated at 200 °C. **b** High-resolution TEM image of Al_2_O_3_/BP sample, same scanned region marked by a *red square* in **a**. The thickness of Al_2_O_3_ and PO_*x*_ layer is 10.7 and 6.1 nm, respectively. **c** Selected area electron diffraction (SAED) pattern for the BP crystalline in **b**

Finally, the interface properties of Al_2_O_3_/BP samples were also characterized by TEM measurements. It can be clearly seen for Fig. 6a that the interfacial PO_*x*_ layer between Al_2_O_3_ and BP was formed during PEALD process with the 20 cycles O_2_ plasma pretreatment. Figure 6b shows high-resolution TEM (HRTEM) image of the Al_2_O_3_/BP sample after the deposition of 100 cycles Al_2_O_3_, same scanned region marked by a red square in Fig. 6a. The thickness of PO_*x*_ and Al_2_O_3_ is 6.1 and 10.7 nm, respectively. It is worth noting that Al_2_O_3_ and PO_*x*_ film is amorphous, while our BP sample is single crystalline which is verified by results of selected area electron diffraction (SAED) pattern, as seen from Fig. 6c. This interfacial layer PO_*x*_ was evidenced by TEM results, indicative of O_2_ plasma penetrating into PO_*x*_ layer and reacting with underlying BP.

## Conclusions

In summary, we have demonstrated the direct growth of Al_2_O_3_ film on BP by using PEALD. The A_1g_ peak of freshly exfoliated BP sample shifts downwards owing to the formation of PO_*x*_ in the BP surface. The uniform Al_2_O_3_ film on BP can be achieved by PEALD with O_2_ plasma and TMA precursors, which may be attributed to the etching and reactivity of O_2_ plasma with BP at high temperatures. The interfacial layer of PO_*x*_ between Al_2_O_3_ and BP was converted into P_2_O_5_ as the deposition temperature increases to 350 °C, revealed by XPS characterizations. These findings provide insightful information on passivation and top-gate dielectric integration for future applications in BP devices.
